# A case study of well child care visits at general practices in a region of disadvantage in Sydney

**DOI:** 10.1371/journal.pone.0205235

**Published:** 2018-10-11

**Authors:** Pankaj Garg, John Eastwood, Siaw-Teng Liaw, Bin Jalaludin, Rebekah Grace

**Affiliations:** 1 Department of Community Paediatrics, Liverpool Hospital, Liverpool, New South Wales, Australia; 2 Specialist Disability Health Team, The Children’s Hospital at Westmead, Westmead, New South Wales, Australia; 3 South Western Sydney Local Health District, Sydney, New South Wales, Australia; 4 Ingham Institute for Applied Medical Research, Liverpool, New South Wales, Australia; 5 School of Women’s and Children’s Health, UNSW, Sydney, Australia; 6 School of Public Health, University of Sydney, Sydney, New South Wales, Australia; 7 School of Public Health, Griffith University, Gold Coast, Queensland, Australia; 8 Department of Community Paediatrics, Sydney Local Health District, Croydon, New South Wales, Australia; 9 School of Public Health and Community Medicine, UNSW, Sydney, Australia; 10 Academic General Practice Unit, Fairfield Hospital, Fairfield, New South Wales, Australia; 11 Faculty of Human Sciences Department: Department of Educational Studies, Macquarie University, Sydney, Australia; Hospital for Sick Children, CANADA

## Abstract

**Introduction:**

Well-Child Care (WCC) is the provision of preventive health care services for children and their families. Prior research has highlighted that several barriers exist for the provision of WCC services.

**Objectives:**

To study “real life” visits of parents and children with health professionals in order to enhance the theoretical understanding of factors affecting WCC.

**Methods:**

Participant observations of a cross-sectional sample of 71 visits at three general practices were analysed using a mixed-methods approach.

**Results:**

The median age of the children was 18 months (IQR, 6–36 months), and the duration of visits was 13 mins (IQR, 9–18 mins). The reasons for the visits were immunisation in 13 (18.5%), general check-up in 10 (13.8%), viral illness in 33 (49.2%) and miscellaneous reasons in 15 (18.5%). Two clusters with low and high WCC emerged; WCC was associated with higher GP patient-centeredness scores, younger age of the child, fewer previous visits, immunisation and general check-up visits, and the solo general practitioner setting. Mothers born overseas received less WCC advice, while longer duration of visit increased WCC. GPs often made observations on physical growth and development and negotiated mothers concerns to provide reassurance to them. The working style of the GP which encouraged informal conversations with the parents enhanced WCC. There was a lack of systematic use of developmental screening measures.

**Conclusions:**

GPs and practice nurses are providing parent/child centered WCC in many visits, particularly when parents present for immunisation and general check-ups. Providing funding and practice nurse support to GPs, and aligning WCC activities with all immunisation visits, rather than just a one-off screening approach, appears to be the best way forward. A cluster randomised trial for doing structured WCC activities with immunisation visits would provide further evidence for cost-effectiveness studies to inform policy change.

## 1. Introduction

Well-Child Care (WCC) is the provision of preventive primary health care services for children and their families. It involves the delivery of anticipatory guidance, developmental screening and surveillance, immunisation, child and family psychosocial assessments and care co-ordination [1, 2]. It aims to support parents’ knowledge about their children’s development, improve parent-child interactions, reduce avoidable hospitalisations and emergency department visits, and has the potential to modify parenting practices in terms of healthcare-seeking behaviour, promotion of positive dietary and feeding practices, and management of common childhood infections [3–7].

WCC is important, as population level studies of early childhood development using the domains of physical health and well-being, social competence, emotional maturity, language, cognitive and communication skills, have identified that more than one-fifth of children are developmentally vulnerable in one or more domain in their first year of formal schooling [8]. In addition, there are several reports that have documented increasing rates of child behavioral problems, developmental delays, and Autism Spectrum Disorders [9–11]. The routine incorporation of developmental screening can significantly increase early identification of developmental delays [12]. Thus, WCC has an important role to play in the identification and referral to appropriate early childhood intervention services that will support families and improve child outcomes [13, 14].

There are several provider and user level barriers in the provision of WCC [15, 16]. A recent population level survey from North America of 34,843 participants has demonstrated that parents’ attitudes and beliefs about having a usual source of care was strongly associated with their children’s receipt of recommended preventive health services [16]. The implication of this research is that access to WCC is driven by ecological variables of service availability, parent awareness and choice of services, and beliefs about their child’s health. It is, therefore, not surprising that vulnerable populations’ with lower health literacy are less likely to access WCC services than the socially advantaged groups [17, 18].

Health professional barriers include organisational factors and attitudes towards prevention, clinical and competing priorities, time, funding models and incentives, the roles of professionals (nurse versus physician providers) and educational and training needs [15, 19, 20]. Physician use of developmental screening tools has remained sub-optimal, but this approach has the potential to improve with additional support and encouragement [21, 22]. Qualitative research has shown that health professionals particularly the General Practitioners (GPs), mostly provide WCC as an opportunistic activity [23]. Thus, alternative innovative models, such as the use of web-based and educator led parent-coach models are explored and studied in recent literature [24].

A search of the SCOPUS database by the authors using an iteratively developed search strategy for studying country based distribution of research on WCC activities revealed that almost three-quarters of published research on WCC comes from North America and the United Kingdom (UK) (S1 Appendix Fig A). Because of this relative paucity of information from Australia, a longitudinal birth cohort ‘Watch Me Grow’ study has recently been conducted in the South Western Sydney (SWS) region of New South Wales. This study confirmed previous international research on factors affecting WCC and developmental screening activities [25, 26]. However, there is an absence of research that directly observes “real life” WCC visits between GP’s and parents with young children in the context of the Australian public health system. Such research can enhance theoretical understanding of the factors affecting WCC, which may further inform interventional and translational studies.

### 1.2. Aims

This study aims to assess the delivery of WCC during GP consultations with young children (less than five years), and understand the factors that enhance or hinder the provision of such services. Specifically, this research was guided by the following questions: (a) How do GPs, parents and children interact together to discuss WCC in the context of the parental reasons for the visit? (b) Are there parent, practice and GP level factors that enhance or hinder WCC?

## 2. Methods

### 2.1. Ethics approval

Ethical approval for the study was obtained from the SWS Local Health District Human Research Ethics Committee (Protocol HREC 13/LLPOOL/265, local project number 13/166).

### 2.2. Study setting

The SWS area ranks low on the Index of Relative Socio-economic Disadvantage with high unemployment rates of 5.2 to 22.3% as compared to the NSW average of 4.7 per cent [27]. The population of this region is relatively young with about 15% of residents being children 0–8 years of age. This is also an area with a large population of people from culturally and linguistically diverse backgrounds, with approximately 34% of residents born overseas [28].

Child health promotion activities are a high priority in the region, with a focus on the availability of universal services, development of targeted services for specific vulnerable groups such as a sustained nurse home visiting program, translation of PEDS to other languages, and access to public funded community paediatric clinics [29].

### 2.3. Study design

A mixed methods critical realist study with a sequential research study design with both qualitative and quantitative studies with parents and professionals was employed to study WCC activities. These studies informed the development of the case studies of the general practices that are described in this paper. The case study design used an embedded approach with concurrent collection of qualitative and quantitative data.

#### 2.3.1. Conceptual and methodological framework for the case study

**Fig 1:**
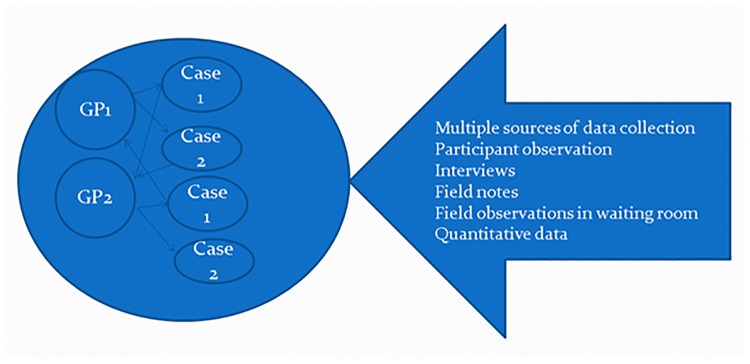
Embedded case study research design.

Two methods were used for data recruitment and analysis: (1) recruitment of practices was done using a purposive approach by addressing the typology of the practice; (2) data analysis unit included both the practice level and the individual GP visits (each participating practice as well as the visit formed a case). This study design in which there is more than one sub-unit of analysis within different contexts is described as an embedded case study design (Fig 1) [30, 31].

We used a realist methodological approach for this work as it addressed the complex ontological and epistemological tussle of tangible *vs*. intangible reality, establishment of regularities established by empirical methods *vs*. knowledge constructed by social interactions, hypothesis testing versus in-depth fieldwork and verification of findings versus interpretation of meaning[30].

Further a critical realist approach helped to view reality as an open system and therefore acknowledged the fallibility of what we considered as real. This approach does not accept empirical observations as the only domain of reality, and includes explanations about how entities are structured, and the conditions that are needed to activate the mechanisms, thereby producing observed or unobserved events [32]. In the context of our case study design WCC is a type of social activity that is delivered in open systems, and mechanisms at hierarchical levels interact during a WCC visit. Thus, a realist framework helped to capture the empirical (from multiple cases and multiple contexts) to actual (what really happens in the human interactions for delivery of WCC) and to a partial extent, understand the generative mechanisms at play during WCC visits [32].

#### 2.3.2. Practice recruitment

Three general practices were identified from the region; two from an electronic Practice Research Network (ePBRN), a group of general practices recruited as an initiative of the University of New South Wales, for improving the data quality of chronic diseases [33]. A third practice was recruited at the suggestion of a GP working at one of the recruited ePBRN practices. The observations at the practices were made between August 2013 and January 2014.

Prior to enrolment, the researcher (first author) introduced himself to the practice staff, including GPs, outlining the overall objective of the research, but without giving many specifics of the research questions. The idea was to capture the interactions between the health providers and parents as they happen in real life. The researcher remained a silent spectator in all the visits therefore causing minimum interference. Written informed consent was obtained from the practice managers, GPs, practice nurses, dental practitioner and parents to audio record the clinical visit. One GP registrar in Practice A and one GP in practice B did not consent the researcher to observe the consultations. One dental practitioner, who was co-located with Practice A, consented to participate in the research.

#### 2.3.3. Data measures

A practice details form was used to collect data on the physical characteristics and resources of each practice. A standardised data collection based on the WCC framework was used for participant observations [34] (S1 Appendix Form A). Observations were also made in the waiting room and extensive field notes were taken.

**Fig 2:**
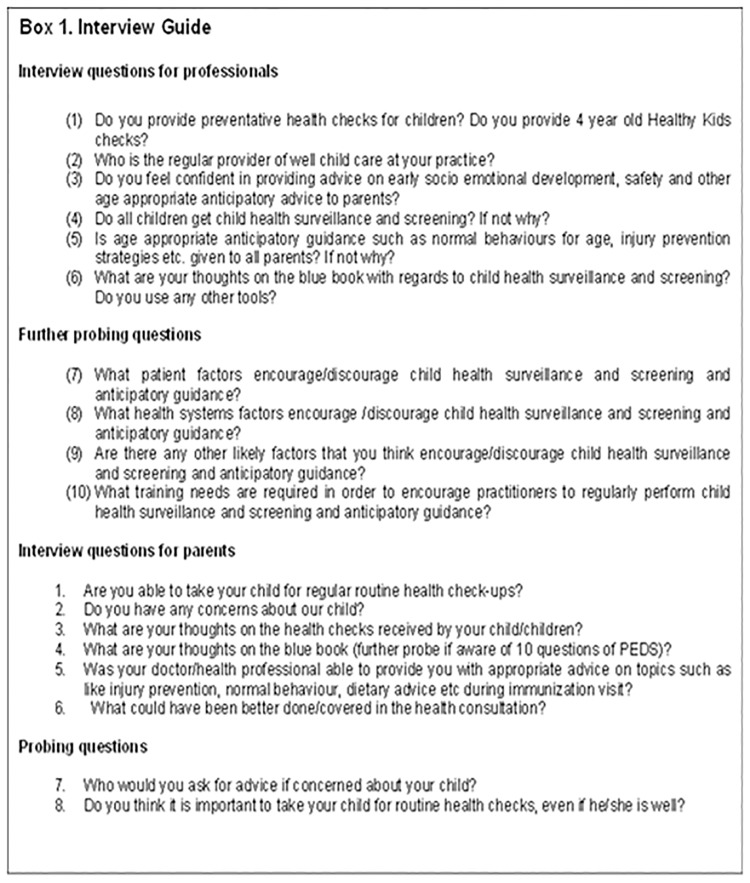
Interview guide.

A selection of parents, GPs and practice nurses, who consented, were also interviewed to collect qualitative data. A structured flexible interview guide was used to facilitate discussion in a non-directive style (Fig 2).

### 2.4. Data analysis approaches

An integrated mixed-methods approach for data analysis was used by connecting and merging both qualitative and quantitative data. This resulted in the convergence of the study findings [35]. Audio recorded files were organised using the NVivo qualitative software, and transcribed by professional transcription services. A sub-section of these transcribed files was verified and cross-checked with the data collected during participant observation. Data analysis started with data collection and stopped on reaching thematic saturation.

#### 2.4.1. Qualitative data analysis

The transcribed files from the audio recordings, field notes, observation forms and interviews provided the qualitative data. Three GPs, two practice nurses, and 34 parents were interviewed during data collection process. Six other GPs provided extensive field notes and during informal conversations which were also recorded. The coding process for the transcribed GP interactions with parents, used a “line by line” and “paragraph by paragraph”, approach to code the components of the medical visits in the broad framework of WCC (S1 Appendix Fig C)[36]. A section of the data was coded at two time points for enhancing intra-coder reliability, and findings were presented in meetings to get feedback and establish trustworthiness in analysis. The author’s clinical and research experiences in community paediatrics, general practice, and public health was acknowledged throughout the process of data collection and analysis.

The proportion of coding for each component of WCC was ascertained using the coding percentages provided by NVivo qualitative software. This approach gave an opportunity to explore which visits had more components of WCC activities embedded in them. This strategy of transforming qualitative data into quantitative data is a well-established method for data integration in mixed-methods research[35].

#### 2.4.2. Quantitative data analysis (Roter’s Interaction Analysis System (RIAS) framework)

In order to study the medical interactions in the visits quantitatively, a Roter’s Interaction Analysis System (RIAS) was used for analysis [37]. RIAS is a previously well described framework in which the contributions of both patients and providers in medical visits are richly elaborated by treating each “utterance” in an interaction as a unit of analysis [37]. It provides categorical coding opportunities in the themes of personal remarks/social conversations, laughing, joking, showing concern or worry, providing reassurance, encouragement, approval, compliment, disagreement, criticism, empathy, legitimizing, partnership statements, showing agreement/understanding, giving orientation, instructions, paraphrase and checking for understanding, asking for opinion, permission, questions (both close & open ended), counselling and encouraging positive behaviour and global affect ratings. The coding framework of RIAS is provided in S1 Appendix Table A. The RIAS categories also provided summary composite scores such as the patient-centeredness scores, which is the sum of biomedical, psychosocial information sharing and rapport building variables between GPs, parents and children, divided by the predominantly biomedical information sharing variables. The higher patient-centeredness scores are achieved by an increased focus on psychosocial information, education and anticipatory guidance during an interaction. 10% of the RIAS files were coded by two independent coders. An average inter-rater reliability between 0.8–0.9 was achieved (S2 Appendix).

#### 2.4.3. Statistical analysis

Descriptive analysis of the demographic and categorical data was conducted. Exploration of patterns was completed using: (1) cross-tab analysis and comparison of proportions using chi-square analysis; and (2) one-way analysis of variance (ANOVA) to explore continuous variables such as the duration of consults, patient-centeredness scores of GPs for visits, previous visits, age of the child, number of previous visits, number of siblings, and categorical variables such as the purpose of visits; for group differences regarding WCC coding percentages.

Regression lines were used to explore the relationships between variables and WCC coding. Multiple regression analysis was also done and for this purpose WCC coding percentages was taken as a dependent variable; maternal country of birth, maternal education status, continuity of the GP provider, number of previous visits at the same practice, age of child, duration of visits, purpose of visit, birth order, GPs years of practice, and patient-centeredness scores were treated as independent variables.

For non-normally distributed data a logarithmic transformation was used. The goodness-of-fit of the model was calculated using “R” analysis, histogram of the standardised residuals and P-P plots, and multi-collinearity was checked using variance inflation factor and tolerance levels (S1 Appendix Fig F). A missing variable analysis showed the completely random nature of the missing values, therefore, a list wise deletion of the cases was done for multiple regression analysis (S3 Appendix). Further, there was no difference in background demographic variables between the missing data to the available data.

A multivariable hierarchical cluster analysis was also done to ascertain if there were clusters of higher or lower WCC visits (i.e. homogenous groups). A squared Euclidean distance was used for distance between all the pairs of cases and clusters using the proximity matrix[38]. Ward’s method of linkage was used to join clusters as this method minimized the total within-cluster variance[39]. The variables were standardised from -1 to +1 to give each variable equal metrics and equal weight. The dendrogram, agglomeration coefficients and scree plots provided the information on relative measure of the magnitude of differences between variables or clusters at each step of the process (S1 Appendix Form D).

The face validity of the clusters was checked using ANOVA and Chi-square to compare how the cases were allocated to the clusters with respect to the potentially important variables.

IBM SPSS Statistics for Windows, version, 24.0. Armonk, NY and MedCalc version 16.8.4, Ostend, Belgium were used for statistical analysis.

## 3. Results

A total of seventy one visits of direct observations with GPs at three practices were completed. Of those clinical visits, eight visits had practice nurse involvement. Fifty visits had good quality audio recordings for RIAS analysis, and complete background demographic data for cluster analysis was available for 44 visits (S3 Appendix Fig A).

### 3.1. Characteristics of the participants

The three recruited practices were different with respect to the number of GP providers, availability of practice nurses, appointment, and billing types **(**Table 1). The demographic characteristics of the participants are presented in Tables 2 and 3.

**Table 1:** Characteristics of recruited practices.

Characteristic	Practice A	Practice B	Practice C
**Type**	Large group >6 GPs (7 GPs and 1 GP registrar)	Small group (3 GPs and one GP registrar)	Solo GP
**Appointment**	Walk in (~60%)Appointment (~40%)	Predominant appointment	Appointment only
**Practice nurse(PN)**	1 (full-time)	0	1 (part-time)
**Immunisations/WCC by practice nurses**	Always	GP	First PN asks questions about WCC, Immunisation by GP
**Facilities**	Pathology, ECG, Oxygen, minor wounds	Minor surgery/wound repairs/ECG/Spirometer	ECG, Spirometer
**Billing**	Fully paid by Medicare	Private billing OOP*	Private billing OOP*
**Child friendly (toys, kids’ books, kids chairs in waiting room)**	No	Yes	Yes
**Relationship of GP with practice**	6 owners, 2 non-owners	3 owners, 1 non-owner	Owner
**Allied Health professionals**	Dentist, Dietician, Podiatrist	1 Psychologist	None

*OOP- out-of-pocket expenses to the families

**Table 2:** Demographic characteristics of the professional participants.

Practice	Profession	Age (yrs) Mean (range)	Gender	Country of birth	Primary language other than English	Years practicing in AustraliaMedian (IQR)	Hours worked per week in the practiceMedian (IQR)	Average child/wk/GPMean (range)	GPs time in the current practice (yrs) Median (IQR)	How often same child seen regularly, self-report by GP?*
**A**	6 GPs		4 Male 4 Female	Australia/NewZealand-1 Others-5	5	11 (6–25)	32 (20–42)	17 (5–30)	2.5 (1.1–7.7)	<25%- 125–50%-351-70%-4
	1 Practice nurse									
**B**	3 GPs	54 (40–60)	2 Male 1 Female	South East Asia-3	2	33 (12–34)	40 (32–40)	15 (10–25)	27 (6.9–30.7)	51–75%-2
										>75%-1
**C**	1 GP 1 Practice nurse	53	1 Male 1 Female	Asia-1 Australia-1	1	21 (14–28)	38	28 (17–40)	13.5 (5–22)	51–75%-1>75%

*This measure is a self-report from GPs and its accuracy could not be verified

**Table 3:** Demographic characteristics of mothers.

Characteristic	N = 71 (%)
**Country/Region of Birth**	
Australia	44 (61.9)
Indian sub-continent	5 (7.1)
Middle eastern	4 (5.6)
South American	2 (2.8)
South East Asian	2 (2.9)
Others	2 (2.8)
Unknown	12(16.9)
**Working status**	
No	18 (25.7)
Yes	44 (61.4)
Unknown	10 (12.9)
**Maternal education status**	
Never attended school	6 (8.4)
Primary school	4 (5.6)
Year 10	6 (8.4)
Year 12	13 (18.3)
Trade qualification	15 (21.1)
Undergraduate degree	10 (14.1)
Postgraduate degree	5 (7.2)
Unknown	12 (16.9)

The median age of children observed in the visits was 18 months (IQR, 6–36 months). There were 29 males and 42 females. The median duration of visits was 13 mins (IQR, 9–18 mins). The median number of previous visits reported by the parents for the same child at the same practice was 6 (IQR, 4–12). There were 56 (85%) standard consultations (<20 minutes duration) and 10 (15%) long consultations (20–40 minutes). The primary reason of visit as stated by the parents included immunisation in 13 (18.5%), general check-up in 10 (13.8%), viral illness 33 (49.2%) and miscellaneous reasons in 15 (18.5%). Overall, there was no relationship between the duration of consults and the purpose of visits (Chi square 3.1, p = 0.37).

### 3.2. Quantitative results

**Fig 3:**
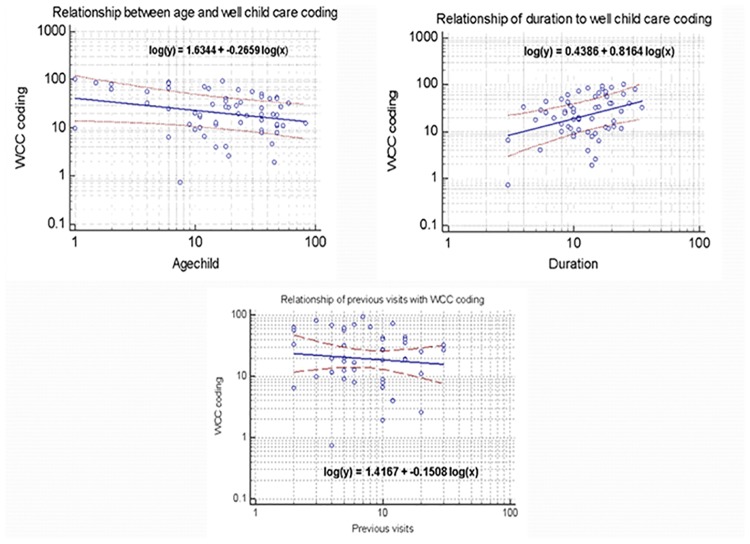
Regression analysis results for Well-Child care coding and important variables.

Regression analysis revealed that greater WCC activities were associated with younger age of the child, longer duration of the visits, and less number of previous visits at the same practice **(**Fig 3). The multiple regression analysis revealed that the visits by Australian born mothers and a longer duration of visit were independently associated with more WCC activity **(**Table 4). This model accounted for about 60% of the variance in WCC activity in the visits.

**Table 4:** Multiple regression analysis.

Variable	Beta	SE	Standardised coefficients	Significance (p-value)
(Constant)	22.690	32.169		.487
Mat birth -Overseas	-18.449	8.192	-.321	.**034**
Mat education level	3.450	9.041	.064	.706
Continuity GP group	.399	12.539	.006	.975
Previous visits	-.895	.557	-.255	.121
Age child (months)	-.321	.249	-.238	.209
Duration (mins)	1.524	.677	.395	.**034**
Purpose of visit	-.029	5.140	-.001	.996
Birth order >1	-3.568	2.630	-.198	.188
GP- Yrs. of practice	.852	.420	.356	.054
Patient centeredness score	23.592	15.809	.212	.149

R for the model-0.77, R-square-0.59,*p<0.05, significant, SE standard error, missing value deleted list wise

**Fig 4:**
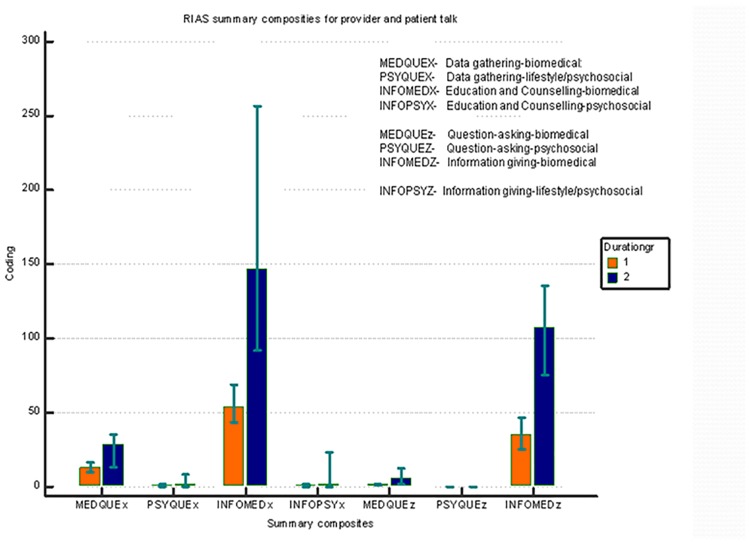
RIAS summary composite results for the medical and WCC visits by parents.

A summary composite analysis of the RIAS coding categories showed that the predominant part of the visits focused on bio-medical information and psychosocial information was sought much less often during the observed interactions **(**Fig 4).

A median GP patient-centeredness score of 0.38 was achieved (IQR, 0.27–0.54). Higher patient-centeredness scores were associated with the longer duration of the consults and greater WCC activity (S1 Appendix).

The immunisation visits had a significantly higher patient-centeredness score (Chi square = 8.5, p = 0.0036).

A hierarchical cluster analysis revealed two distinct clusters; one with higher and one with lower clusters for WCC activities. The cluster with higher WCC had higher patient-centeredness scores during the interactions, more first born children, younger infants, and those presenting for immunisation or general check-ups as a primary purpose of their visits **(**Table 5).

**Table 5:** Univariate analysis of demographic and practice level characteristics of GP visits according to clusters.

	Mean (SD) Ptcentscores	Mean(SD) Duration (min)	Mean (SD) Previous visits	Age (months) Median (IQR)	Mean (SD) WCC coding %	Siblings Median (IQR)	Solo GP *vs*. GP in group practices	Maternal educational status-University N (%)	Country of birth Australia N (%)	Immunisation & general check-up visits N (%)
**Low WCC Cluster** **(n = 32)**§	0.42(0.2)	13.1 (7.1)	10(7)	19(15–36)	17.8 (11.7)	1(1–2)	1 (3%)	8/32	23(71)	5(15.6)
**High WCC Cluster** **(n = 12)**§	0.61 (0.4)	17.4 (4.6)	3.5(2.7)	6 (2–14)	71(15.2)	0(0–1)	4 (33.3)	8/12	11(91.6)	9(75)
**p-value**	0.04*	0.06	0.003*	0.0017*	<0.0001*	-	0.006*	0.5	0.16	0.002*

^§^ Data is analysed for visits where complete data for all variables was available, Means compared using t-test, comparison of proportions test (Fisher exact or Chi Square), Medians compared using Mann Whitney test,

*p-value <0.05 significant,

WCC-well child care, Ptcentscores-patient-centeredness scores, GP- General Practitioner

### 3.3. Qualitative results

#### 3.3.1. Health professional opinions- competing priorities and system issues

All GPs and practice nurses who participated in the study expressed the importance of WCC but acknowledged that it is not done often: “*in reality we don’t do much preventive activities as we don’t have time*, *but we do ask parents questions and do observations*, *and if there are concerns we do the needfu*l” [GP1]. Another GP implied WCC as part of GP training and (implicit) practice: “*we don’t need to worry about Medicare Healthy Kids Check if all of us do what we are trained for*” [GP7].

Other health professionals expressed that prompts are needed more explicitly in the records for both parents and providers: “*little awareness which is put forward regarding PEDS (developmental screening tools) in the Personal health Records*” [GP5]. It was also reported that a lot of discussion on WCC is “*by observation and all conversations don’t get recorded*”[GP6].

Some commented that funding systems don’t support screening and WCC activities: “I *don’t think Medicare is happy for us to charge Level C (long) consultation for screening activities*”[GP].

Some reported that it will be good to “*have* [the] *practice nurse doing these activities for us*” [GP6]. Personal Health Records were often used to “*record immunisations*, *height and weight*” [PN1]. Parents sometimes forgot to bring their personal health record and were reminded by the GPs in their visits of the importance of bringing these as a prompt for WCC discussions: *“Always remember to bring your blue book when you visit next time” [GP6]*.

A dental practitioner located within one general practice reported that preventive dental checks don’t happen as “*parents come only when there is a problem*” [D1].

#### 3.3.2 Parental choice of services

Parents often made the choice to access services for health checks based on (a) **convenience**: *“I stopped going to Fairfield Community health centre…and has been coming to this practice (GP) as it is convenient*” [P2]; (b) **the purpose of their visit**: “*I go to the child nurse if I have questions about his sleeping and behaviour*, *if he has a cold I will come here (GP)*” [P12]; (c) **their prior experiences with the health professional**: “*I don’t go to midwife and nurse as they gave a lot of conflicting information*” [P11]; (d) **approach of the health professional to their children**: “*(GP) is fantastic*, *my kids just love him and want to keep coming again and again*”[P8]; (e) **after hours availability**: “*I come here as doctors are available afterhours*, *even though the surgery is bit away from my house*” [P10]; (f) **long term relationship with the GP**: “*I have been visiting this practice since I was a baby and always come here*” [P2]; and (g) **their beliefs about whether or not their child needs to see a health professional**: “*I don’t see a need to go to child and family health nurse as he is well*” [P8]. Parents also reported lack of awareness of questions in the personal health record, “*I am not aware that there are questions in the Blue Book*” [P23].

#### 3.3.3. Eliciting and clarifying parental expectations during interactions with health providers

A qualitative analysis of the encounters revealed that parent’s expectations were an important mechanism for WCC activities. Where a parent raised a concern or a WCC topic, these were often addressed appropriately and reassurance was provided. There was also a sense that the bio-medical model, especially diagnosis is central to general practice. The initial conversation of a GP with the mother of a 4 year old child below is an example how it may require an effort to adopt the health promotion/preventive approach of WCC during some interactions:

“Doctor: What’s the problem with him?

*Mother: Just check-up, normal check-up*.;

*Doctor: Normal check-up, is there something you worry about*?

*Mother: No his height, weight*;

*Doctor: Oh! You want to check these things”*;

*Mother: I don’t know, comparing to his brother*-;

Doctor: So he is smaller than his brother?

*Doctor (after taking anthropometric measurements): It’s (height, weight) all right, according to the charts*;

*Doctor: Is he eating well*?

*Mother: He doesn’t have an appetite*;

Doctor: We can check him in one to three months. He is active. He is happy. He is running. Is he talking?”

[Practice A]

During some interactions those development questions were asked that were related to the diagnosis and assessment of the presenting health problem. This is illustrated in the transcript excerpt below relating to a 3 year-old girl presenting with a suspected urinary tract infection.

“*Doctor*: *She is still in nappies*, *isn’t she*?

Mother: No

Doctor: She is not?

*Mother*: *No*, *she has not been in nappies for a long time*”.

[Practice A]

Growth and development questions were sometimes related to the basic information for management of health problems. For example, child weight often came up in the context of interactions for common illnesses where a medication was prescribed by weight:

“*Doctor*: *So what’s her weight*?

*Mother*: *She is eleven and a half kilos*,

*Doctor*: *Did you check her recently*,

*Mother*: *Uh well*…

*Doctor*: *Let me just weight her again*”.

[Practice A]

Some GPs have a style that provided opportunities for informal conversations with children; particularly during the four year immunisation visits. This type of approach helped in ascertaining language development of children. A referral to a specialist was provided after acknowledging parental concerns regarding their child:

“*Doctor*: *I like your shoes, you got nice shoes*.

*Child: Thank you*.

*Doctor: They match very well with your skivvy*.

*Child: What is skivvy*?

*Doctor: Your top, and match very well with your shoes*.

*Child: Now they are not good, they had no black laces*.

*Mother: I am worried about my son as well*.

*Doctor: What*?

*Mother*: *He has always been kind of clumsy*. *He starts school next year and* [the preschool teachers wonder] *whether he should be referred*, *as he likes independent play as opposed to playing with other children*. *He doesn’t talk much at school*.

*Doctor: At preschool quiet and at home he is chatty*.

*Doctor: Tell me daddy’s name*.

*Child*: [gives his name]

*Doctor: I might get you to see a paediatrician*.”

[Practice B]

At the practices where practice nurses provided immunisation, there were opportunities given for an assessment of age-appropriate hand-eye coordination and cognitive skills,:

“*Nurse*: *What’s your favourite food*?

Child: Apple

*Nurse: What colour is that*?

*Child*: *Green*

*Nurse: Are you able to draw a tree over there*?

Child: Pen

*Nurse: Keep going, yeah What about all the braches and the leaves*?

*Nurse: Can you draw a circle next to the tree*?

*Child: Yes, here*”

[Practice A]

During visits for young children where there were concerns about minor viral illnesses, focused education and counselling was provided to parents for recognition of sickness. WCC activities were also discussed as illustrated by the following transcript excerpt from a mother presenting with a five week old infant with upper respiratory symptoms:

“*Mother*: *I am not sure, there are viruses going around in this area but he just sounded awful last night, he couldn’t sleep*…

*Doctor: Is there anything coming out of his nose*?

*Mother*: *I saw some* [nasal discharge]. *He just could not breathe for a few days now*.

*Doctor: So are you breastfeeding him*?

*Mother: Half and half, but I do try and do it a few times (every day)*.

*Doctor: Saline (drops) for the nostrils. Do it as you feed him… If he spikes a temperature, you are noticing breathing issues, Becoming very lethargic, you notice a rash, start vomiting, anything, Any red flags, please come back*.

*Mother: What about the wind? Can I give Infants Friend*?

*Doctor: I mean all kids (up to) the age of 12 weeks will have the same thing, so that’s (colic behaviour) is normal*”

[Practice C]

#### 3.3.4. Other participant observations

Developmental screening tools were not used in any visits and missed opportunities were noted in provision of guidance on injury prevention, obesity and feeding practices. Case analysis of the visits revealed that immunisation visits and some visits for minor viral illnesses had higher patient-centeredness scores and more WCC activity **(**Table 6).

**Table 6:** Characteristics of GP visits with higher patient centeredness scores and well child care activities.

Ptcent score	Duration of visit(mins)	Purpose of visit	Age (months)	WCC coding percentage	Practice	Nurse	Prior visits	Continuity group	Siblings
1.07	18	Immunisation	2	62.5	Practice B	No	2	>75%	3
1.17	10	Immunisation	19	26.5	Practice B	No	10	50%	1
1.44	17	Immunisation	18	93.8	Practice C	Yes	7	>75%	1
1.71	11	viral illness	-	72.3	Practice C	No	12	>75%	2
0.88	13	Immunisation	2	85.4	Practice C	No	-	>75%	1

## 4. Discussion

The study elaborates on the lived experiences of parents and GPs and their interactions relating to Well-Child Care topics as well as on education and counselling pertaining to the primary reason for the GP visit. The profile of visits, in terms of the duration of the consults and the proportion of long and standard consultations, was consistent with the population based data on GP visits in Australia [40].

### 4.1. Propositions for well child care

Two important propositions for WCC activities emerged from the interactional data analysis in this study: (1) *Context—*parent attends for immunisation; *Mechanisms–*Parent asks questions on general check, doctor aware and knowledgeable, practice nurse support, time availability; *Outcome—*anticipatory guidance and WCC provided, (2) *Context*—Parent presents with minor illness; *Mechanisms—*Doctor does a focused history and exam and provides information, education and counselling on the recognition of sickness, opportunistically notes language delays or questions development, comments on growth, may have a rushed approach and importance not given to these issue because of not being the primary reason of visit, *Outcome*—missed opportunity for anticipatory guidance, follow up may or may not be planned.

### 4.2. Qualitative

The results from the study confirmed prior qualitative data that WCC and other preventive activity was predominantly based on observation of the child and provided opportunistically [23]. There are obvious reasons for this including time constraints, and duplication for writing this information in the Personal Health Records and the general practice software.

No screening tools were used by GPs for ascertaining developmental progress in this study and the Blue Book was used for recording immunisations and anthropometry only. This is somewhat contrary to a survey of GPs where two-thirds of practitioners reported using a standardised tool in their clinical practice for developmental screening [41].

In the current observational study, when parents raised concerns about their child’s development, appropriate advice and/or referral were provided by GPs in most interactions. In GP visits for the one-off Healthy Kids Check (HKC) prior to school entry (that stopped being reimbursed in November 2015, and was rescinded in July 2016), there were several opportunities for the family to discuss WCC activities. This is in congruence with the positive impact of the HKC visit on the management plan for 3–11% of children at two general practices in Queensland, that is likely to translate to a significant number of children at the national level [42].

The purpose of the visits was predominantly biomedical particularly where parents presented with children for minor illnesses [43]. However, it is possible that the psychosocial conversations happened during prior or later general practice visits with parents that were not directly observed.

In other qualitative studies it has been shown that parents form a working diagnosis with their family when concerns arise regarding their child’s health and parents make many more visits to their GP with their first born children [18].

The finding that a solo GP with a practice nurse achieved a higher patient- centeredness score is possibly explained by an increased continuity of care by the same provider and suggests that the motivation, training and communication style of the physician are also important factors affecting WCC [43].

### 4.3. Quantitative

In visits with a primarily biomedical focus, patient-centeredness scores and WCC activity were lower, indicating the need for a communication style by practitioners that balanced psychosocial and biomedical topics during the visits [44].

Longer duration of consults was associated with Well-Child care activity in our study. This finding is well-demonstrated in a population based North American National Survey of Early Childhood Health where longer visits were associated with more anticipatory guidance, psychosocial risk assessment and higher family-centered care ratings [45]. It should also be noted that the use of validated screening tools can also increase parent-physician communication about developmental concerns without necessarily increasing the duration of consults [46].

There was a trend for immunisation visits to be longer particularly in visits where practice nurses were involved in doing the four year HKC check. This gave more time for families to discuss issues, and lends support to the nurse practitioner led model of WCC care in the general practice context. The recent findings from the primary health care network surveys in Australia highlight that the number of practices with a practice nurse is increasing and practice nurses have expressed interest to play a key role in WCC activities [47].

The study finding that young children with a mother born overseas receive less WCC activities t highlights the challenges associated with the provision of health services for culturally and linguistically diverse mothers. This issue has been explored in greater details in previous studies[48].

### 4.5. Strengths and limitations of the study

To the best of our knowledge this is the first study from Australia that analyses WCC activities at general practices based on direct observations of the GP visits. The information is of value for stakeholders, administrators and policy makers in the context of the Australian health system. Although not generalisable, these findings are likely to be transferable to similar settings.

The most vulnerable families may not have been captured in our study and further studies need to explore the issues highlighted in our study with the most disadvantaged groups of parents.

It could be argued that some GPs may have asked more or less questions on WCC activities if the researcher was not present. However, this was unlikely as the flow of conversation between the GP and parents that was evident in the audios and transcribed data, showed no interference from the researcher, providing confidence in the authenticity of our data. Further, the qualitative analysis was complemented by the RIAS coding that was verified by independent coders separate from the researcher doing the data collection. This mixed methods analytical approach provided credible evidence of the main issues in the delivery of WCC during visits.

Engagement of the parents with GPs was not specifically scored in the current study. This is a known variable that can affect their approach to WCC.

The influence of previous visits and the continuity of care with the same provider or with the same practice could not be ascertained as longitudinal visits for the same patient was not possible. Rather a cross-section of visits was studied. Although this factor did not emerge as a major variable in quantitative analysis it does warrant further confirmation in larger studies. The continuity of practitioner in the observed visits was based on GP self-report and its accuracy could not be verified.

The finding of utility of practice nurses as a strategy for enhancing WCC need more work as only a small proportion of observed visits in the current study had a practice nurse involvement.

The study finding of a solo GP being able to do more WCC activities is not generalisable as there only a single solo GP setting was recruited for the study. Larger studies are required to explore this potentially important factor.

For infants presenting for immunisation qualitative research has highlighted that GPs may not necessarily ask many WCC questions during these visits [17]. It is possible that the conversation about WCC may differ for different immunisation visits at different ages. This factor could not be explored in this study due to limitations of sample size.

The precision of calculated statistics is affected by the missing values (S3 Appendix). However, this is not a major limitation as missing values were completely randomly distributed and not systematically different from the analysed cases.

### 4.6. Implications for future studies

There is a need for cost effectiveness studies using an outcomes framework for routine incorporation of screening and surveillance activities during immunisation and Well- Child care visits in the context of the Australian public health system. This study was done when the Medicare funded four year HKC was still active. Further cohort studies and randomised controlled trials are required to assess if the immunisation visits have changed in terms of WCC information provided to the families, pattern of Medicare Benefits Schedule claims and duration of visits since the cessation of HKC program.

There is also a need to research if linking web based developmental screening questions to GP practice software can assist GPs in identification of children with developmental problems. This may be a strategy as this can potentially reduce their administrative work load of data entry into their practice systems.

There is in general a knowledge gap regarding the patterns of cost for Medicare consults and duration of consults with regards to immunisation visits. Cost-effectiveness studies into schedule items numbers claimed by Australian GPs to provide immunisation and general check-up visits would provide support to the introduction of specific Medicare item numbers to encourage WCC activities including the use of standardised tools in general practice [49].

It is also important to research funding mechanisms for GPs from other comparable health systems such as salaried models, annual consolidated fees for each enrolled child in the practice, performance and child outcomes based payments (for example, proportion of toddlers identified with developmental delays linked with early intervention services at a practice level) and understand its relevance to funding Australian GPs for WCC. Future research should explore practice nurse led models of WCC as well as integrated models of care where GPs, practice nurses, paediatricians and CFHNs share data in real time regarding their WCC activities. Educational activities and research on physician communication styles to increased family and patient centred care are also needed.

### 5. Conclusions

Well-Child care is delivered in a significant proportion of GP visits for children. The use of screening tools is infrequent and GPs rely on observations and parental concerns to identify children with developmental delays. There are two distinct clusters of WCC activities within GP visits and younger children and Australian born mothers are more likely to receive this form of care. More studies are required to ascertain how GPs can be supported to use screening tools for WCC surveillance activities during immunisation visits. Longitudinal cohort study and randomised controlled trials of children attending general practice would provide more robust information to guide the development of policies for WCC in Australian general practice.

## Supporting information

S1 Appendix Box A: Australian Public Health System and Training of health professionals in WCC Fig A Country wise publications on well child care activities Fig B NSW Ministry of Health model pathway for child health and development assessment and referral Fig C: Coding categories under the well child care framework Form A: Data collection form Form B: Waiting room form Table A: Roter’s Interaction Analysis System coding framework Fig D Dendrogram and scree plots from hierarchical cluster analysis with practice as unit of analysis for WCC coding Fig E. Relationship with patient centeredness scores with duration of consult and WCC coding Fig F. Histogram and normal P-P Plot of standardised residual dependent variable.
(DOCX)

S2 Appendix Roter’s interaction analysis system, coder reliability. (DOCX)

S3 Appendix Fig A Flow chart of Data loss Missing Variable Analysis.
(DOCX)

S4 Appendix De-identified RIAS raw data. (XLSX)

## Abbreviations

CFHN: Child and Family Health Nurse
GPs: General Practitioners
HKC: Healthy Kids Check
PEDS: Parents Evaluation of Developmental Status
RIAS: Roter’s Interaction Analysis System
SWS: South Western Sydney
WCC: Well Child Care
