# Supplementary material for: A case study of well child care visits at general practices in a region of disadvantage in Sydney
Source: PLoS One. 2018 Oct 11;13(10):e0205235. doi: 10.1371/journal.pone.0205235 (PMC6181326; doi:10.1371/journal.pone.0205235)
Supplement: S1 Appendix — Box A: Australian Public Health System and Training of health professionals in WCC Fig A Country wise publications on well child care activities Fig B NSW Ministry of Health model pathway for child health and development assessment and referral Fig C: Coding categories under the well child care framework Form A: Data collection form Form B: Waiting room form Table A: Roter’s Interaction Analysis System coding framework Fig D Dendrogram and scree plots from hierarchical cluster analysis with practice as unit of analysis for WCC coding Fig E. Relationship with patient centeredness scores with duration of consult and WCC coding Fig F. Histogram and normal P-P Plot of standardised residual dependent variable. (DOCX) [file pone.0205235.s001.docx]

# Box A Australian Public Health System and Training of health professionals in WCC

# 1.1. Australian health services context

Australia has a highly developed primary health care system delivered through the federally funded Medicare Benefits Schedule program (a government insured program for the delivery of primary and specialist services). WCC is provided by two sets of providers; firstly, by child and family health nurses (CFHNs) through State funded early childhood health centers; and secondly, by GPs in private clinics, where the federal Medicare program subsidises the cost of doctor visits. There are variations between states of Australia in the structure and usage of child and family health nurses for WCC activities.

## 1.1.1. New South Wales health services context

In the Australian state of New South Wales, a Personal Health Record for infancy and early childhood, commonly referred to as the “Blue Book”, is recommended by the Ministry of Health as an important tool for delivery of WCC activities (S1 Appendix Fig.2). The Personal Health Record is provided to parents at their child’s birth along with information about the recommended health checks and this is reinforced during universal health home visit and/or at the time of a visit to an early childhood health clinic. The universal health home visit was initially envisioned to be provided to all families within 14 days but evolved into a targeted approach for vulnerable families within SWS due to staffing constraints.

The Personal Health Records has various checklists and prompts for primary health providers to guide and facilitate the discussion of WCC, and at the time of the current study, it also incorporated the PEDS (Parents Evaluation of Development Status), a validated parental led primary health developmental screening tool. Parents are encouraged to complete the PEDS in the Blue Book and discuss any concerns with nurses or GPs during health checks or immunisation visits. The parents voluntarily choose between GPs and nurses and it is known that culturally and linguistically diverse families attend GPs more than nursing services. However, prior studies also indicates that these parents are more likely to report that their concerns are not adequately acknowledged or addressed, and there are gaps in the sharing of health information across sectors.

## 1**.1.2. Training and systems used by nurses and GPs for WCC**

Developmental screening tools are routinely used by CFHN’s in Australia, and a plan of action for review or referral to GPs and other services as needed are documented in the Personal Health Record. However, population-level coverage of health visits delivered by CFHN’s has remained sub-optimal in NSW, where approximately 50% of children between 0 to 11 months, and 35% of children between 1 to 4 years of age, access child and family health nursing services. A population-based study of general practice visits in Australia has demonstrated that for every 100 visits to GPs, four visits are made for immunisation for pre-school children. Hence, GPs could be in a unique position to deliver WCC. Universal child development screening activities are, however, ‘optional’ and do not attract any specific funding or incentives. Another approach in the recent past (2008-2015) employed by the federal government to encourage WCC by Australian GPs was a publically funded, national ‘one-off’ Healthy Kid’s Check (HKC) for four year old children. The HKC focused on a number of domains of well-child visits including developmental screening, but was discontinued to be reimbursed since November 2015. There were cost implications for the families as some general practices had to charge above the Medicare scheduled fees to bear administrative costs.

Training for GPs for WCC in Australia includes online modules linked to written guidelines for preventive health and developmental activities. GPs are also encouraged to consider the use of developmental screening tools such as PEDS, or an alternative approach of ‘red flags’ for developmental milestones, as a means of identifying developmental delays and ensuring early referral.

**Fig A. Country wise publications on well child care activities**


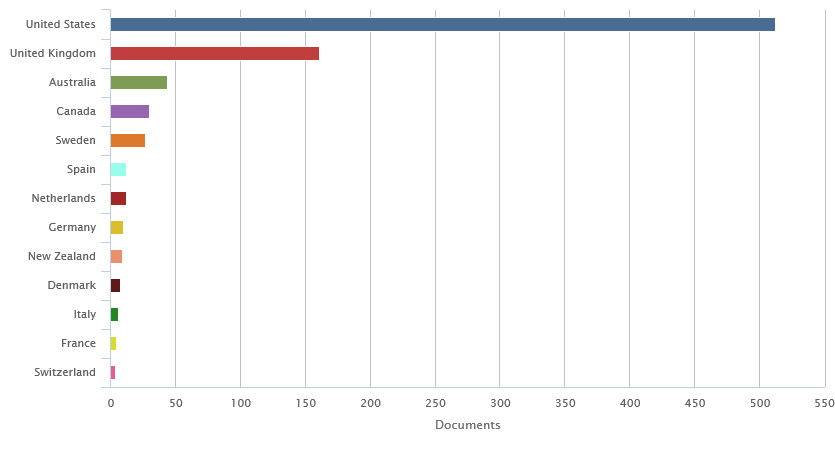


Search strategy (1963-2014), “Child Health surveillance” OR “Child Health promotion” OR “Well Child Care” OR “Preventive Care” AND “Children”

**Fig B. NSW Ministry of Health model pathway for child health and development assessment and referral**

**
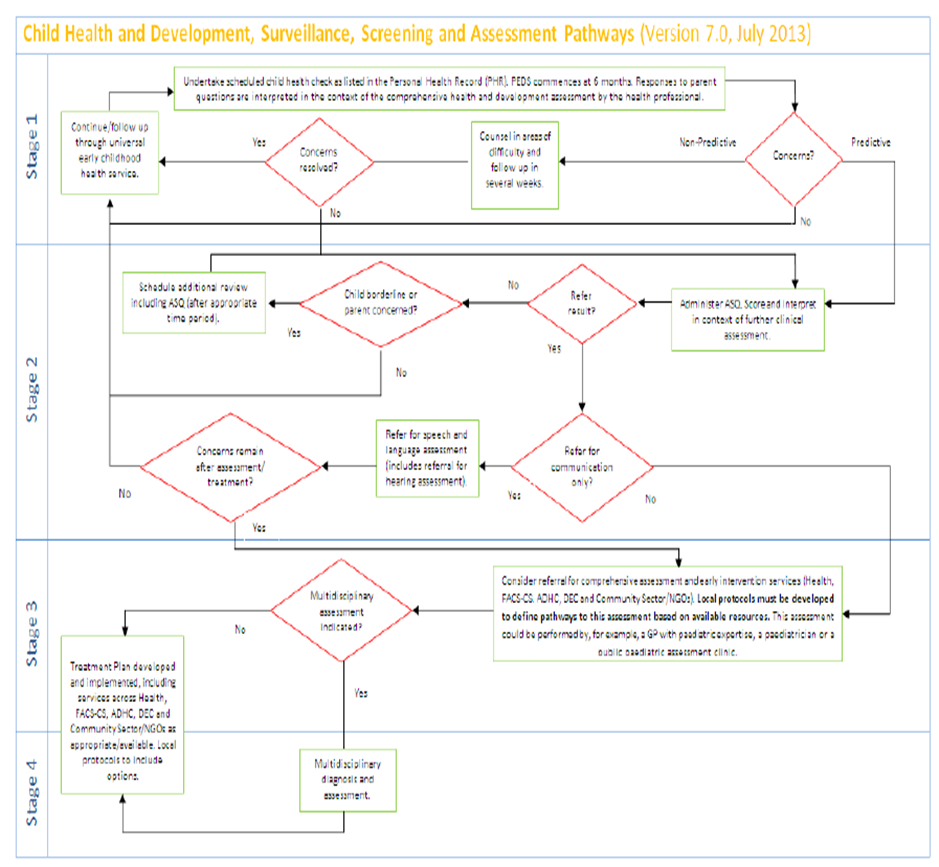
**

**Fig C: Coding categories under the well child care framework**

**Form A. Data collection form**

**Clinical Observation Form**

**Practitioner ID # ____ Questionnaire #code.................................**

**Date................................................**

**Please use the following key for filling the observation form**

(_√_ = Yes; _X__ =No; _√_ pr – if raised by parent; _√_ dr – if by doctor/clinician)

__ Male __ female, __age of child (yrs/months)

_________ start time _________ end time (time duration...................mins)

___ Was there an open-ended start?,___Were parents’ concerns addressed?

**START POINT CLIENT PROFESSIONAL INTERACTION**

| **Rapport** | **Mood** | **Engagement** |
| --- | --- | --- |
| **Professional** | | |
| 1 2 3 4 5  Cold Neutral Warm | 1 2 3 4 5  Cold Neutral Warm | 1 2 3 4 5  Cold Neutral Warm |
| **Client** | | |
| 1 2 3 4 5  Cold Neutral Warm | 1 2 3 4 5  Cold Neutral Warm | 1 2 3 4 5  Cold Neutral Warm |

**Please tick if the following information was recorded or discussed**

| ___ length/height taken___ neck ___ comment | ___ chest ___ comment |
| --- | --- |
| ___ heart ___ comment | ___ abdomen ___ comment |
| ___ genitalia ___ comment | ___ pulses ___ comment |
| ___ arms ___ comment | ___ legs ___ comment |
| ___ neuro ___ comment | ___ discuss exam/other during? |
| _______ min/sec of Physical Exam duration | _______ min/sec discuss PE after exam duration |
| ___ observe parent/child ___ comment |  |
|  | ___ weight taken___ Naked / Clothed partly / Fully (tick appropriate) |
| ___ OFC | _______ growth chart review done |
| ___ vision ___ eyes ___ ophthalmoscope __(used/considered)comment | ___ hearing ___ ears _____ otoscope _____(used/considered)comment |
| ___ HR ___ RR | ___ BP ___ Manual / Automatic |
| ___ screening questionnaire(s) | ___ reviewed? |
| ___ discussed? | ___ child input? e.g. drawing___comment |
| ___ head ___ comment | ___ oral ___ comment___ throat ___ comment |
| **ANTICIPATORY GUIDANCE** | |
| ______ growth __ pr __ dr ______ advice | |
| ______ nutrition/diet ?s __ pr __ dr ______ advice | |
| ______ feeding ?s __ pr __ dr ______ advice | |
| ______ bowel/toilet ?s __ pr __ dr ______ advice | |
| ______ sleep ?s __ pr __ dr ______ advice | |
| ______ vision ?s __ pr __ dr ______ advice | |
| ______ hearing ?s __ pr __ dr ______ advice | |
| PEDS questions asked______ __ pr __ dr ______ advice | |
| ______ gross motor ?s __ pr __ dr ______ advice | |
| ______ fine motor ?s __ pr __ dr ______ advice | |
| ______ language ?s __ pr __ dr ______ advice | |
| ______ Social dev. ?s __ pr __ dr ______ advice | |
| ______ behaviour ?s __ pr __ dr ______ advice | |
| ______ discipline ?s __ pr __ dr ______ advice | |
| ______ violence ?s __ pr __ dr ______ advice | |
| ______ safety (car seat, sleeping position, pool fencing, helmets, safe play areas, fall prevention, sun safety, choking) ?s __ pr __ dr ______ advice | |
| ______ Injury prevention (watching child near water, cleaners, chemicals, medications out of reach) s __ pr __ dr ______ advice | |
| ______smoke free home__ pr __ dr ______ advice | |
| ______ oral health ?s __ pr __ dr ______ advice | |
| ______ phys. activity ?s __ pr __ dr ______ advice | |
| ______ media/screen ?s __ pr __ dr ______ advice | |
| ______ literacy ?s __ pr __ dr ______ advice | |
| ______ day care/preschool/ school ?s __ pr __ dr ______ advice | |
| ______recognition of illnesses __ pr __ dr ______ advice | |
| FAMILY SITUATION/PSYCHOSOCIAL | |
| __ housing issues __ others__ | |
| ______ family/support (DV/other stressors)?s __ pr __ dr ______ advice | |
| ______ mother well ?s __ pr __ dr ______ advice | |
| ______ emotion/temper ?s __ pr __ dr ______ advice | |
| ______ mental health ?s __ pr __ dr ______ advice | |
| ______ social/peers ?s __ pr __ dr ______ advice | |
| ______ community supports ?s __ pr __ dr ______ advice | |
| ______ immunization ?s __ pr __ dr # given ___ | |
| ______ Rx | |
| ______ blood/urine tests | |
| ______ other tests | |
| ______ referrals | |
| MISCELLANEOUS___Time spent in clinical documentation on computer | |
| ___attempts engagement while clinical documentation | |
| ___any specific context factor which could let doctor to explore less (such as knows family well, child’s behavior challenging) | |
| ___child addressed, provided opportunity to talk (if applicable) | |
| ___ turn taking available child or focus the child during interaction (minimal, some, adequate) | |

BLUE BOOK USED ______ Purpose used______

**END POINT CLIENT PROFESSIONAL INTERACTION**

| **Rapport** | **Mood** | **Engagement** |
| --- | --- | --- |
| **Professional** | | |
| 1 2 3 4 5  Cold Neutral Warm | 1 2 3 4 5  Cold Neutral Warm | 1 2 3 4 5  Cold Neutral Warm |
| **Client** | | |
| 1 2 3 4 5  Cold Neutral Warm | 1 2 3 4 5  Cold Neutral Warm | 1 2 3 4 5  Cold Neutral Warm |

**Table 1**

**Form B. Waiting room form**

**DATE Time and Period of observation**

**CHILD AND FAMILY PRACTICE PARAMETERS**

Any separate play area........................................................................

Toys in the waiting rooms...................................................................

**Type hard versus soft**

Observe children activities in the play area.........................................................................................................................................................................................................................................................................................................................................................................................................................................................................................................................................................................................................................................................................................................................................................................................................................................................................................................................................................................................................................................................................................................................................................................................................................................................................................................................................................................................................................................................................................................................................................................................................................................................................

**ENGAGEMENT OF RECEPTIONS WITH PATIENTS**

Welcoming with smile

Neutral

Negative

Answers to questions of staff in a engaging way

**ENGAGEMENT OF THE DOCTOR WITH THE CLIENT AT RECEPTION**

Introduces

Eye contact

Walks away after calling name with much engagement

Directs patient to the room along with him

**OBSERVATIONS OTHER GENERAL ACTIVITIES**

.....................................................................................................................................................................................................................................................................................................................................................................................................................................................................................................................................................................................................................................................................................................................................................................................................................................................................................................................................................................................................................................................................................................................................................................................................................................................................................................................................................................................................................................................................................................................................................................................................................................................................................

**OBSERVATIONS ACTIVITIES OF PRACTICE NURSES**

**------------------------------------------------------------------------------------------------------------------------------------------------------------------------------------------------------------------------------------------------------------------------------------------------------------------------------------------------------------------------------------------------------------------------------------------------------------------------------------------------------------------------------------------------------------------------------------------------------------------------------------------------------------------------------------------------------------------------------------------------------------------------------------------------------------------------------------------------------------------------------------------------------------------------------------------------------------------------------------------------------------------------------------------------------------------------------------------------------------------------------------------------------------------------------------------------------------------------------------------------------------------------------------------------------------------------------------------------------------------------------------------------------------------------------------------------------------------------------------------------------------------------------------**

**OBSERVATIONS ACTIVITIES OF OTHER STAFF**

**---------------------------------------------------------------------------------------------------------------------------------------------------------------------------------------------------------------------------------------------------------------------------------------------------------------------------------------------------------------------------------------------------------------------------------------------------------------------------------------------------------------------------------------------------------------------------------------------------------------------------------------------------------------------------------------------------------------------------------------------------------------------------------------------------------------------------------------------------------------------------------------------------------------------------------------------------------------------------------------------------------------------------------------------------------------------------------------------------------------------------------------------------------------------------------------------------------------------------------------------------------------------------------**

**OBSERVATIONS OF ACTIVITIES OF PATIENTS**

- Focus if they are reading health promotion materials
- Focus on average waiting times of patients
- Focus on doctor was late to start clinic or has to interrupt his clinic for reasons other than consultation
- Punctuality of patients, no shows, walk ins, presence of companions, service time (total time a doctor is engaged with one patient and not able to see another patient)

-------------------------------------------------------------------------------------------------------------------------------------------------------------------------------------------------------------------------------------------------------------------------------------------------------------------------------------------------------------------------------------------------------------------------------------------------------------------------------------------------------------------------------------------------------------------------------------------------------------------------------------------------------------------------------------------------------------------------------------------------------------------------------------------------------------------------------------------------------------------------------------------------------------------------------------------------------------------------------------------------------------------------------------------------------------------------------------------------------------------------------------------------------------------------------------------------------------------------------------------------------------------------------------------------------------------------------------------------------------------------------------------------------------------------------------------------------------------------------------------------------------------------------------------------------------------------------------------------------------------------------------------------------------

**OBSERVE THE PHYSICAL CHARACTERISTICS OF THE PRACTICE**

-------------------------------------------------------------------------------------------------------------------------------------------------------------------------------------------------------------------------------------------------------------------------------------------------------------------------------------------------------------------------------------------------------------------------------------------------------------------------------------------------------------------------------------------------------------------------------------------------------------------------------------------------------------------------------------------------------------------------------------------------------------------------------------------------------------------------------------------------------------------------------------------------------------------------------------------------------------------------------------------------------------------------------------------------------------------------------------------------------------------------------------------------------------------------------------------------------------------------------------------------------------------------------------------------------------------------------------------------------------------------------------------------------

**Table 1. Roter’s Interaction Analysis System coding framework**

| **ALL “PROVIDER” TALK (i.e., all CODED talk, by primary provider, to parent and child)** | **ALL “PATIENT” TALK (i.e., all CODED parent and provider):** |
| --- | --- |
| - **Data gathering-biomedical:** MEDQUEX=CMEDX+CTHEX+COTHX+OMEDX+OTHEX+OOTHX+BIDX+CAGDEX+OAGDEX - **Data gathering-lifestyle/psychosocial:** - PSYQUEX=CLSPSX+OLSPSX+CAGSOX+OAGSOX - **Education and Counseling-biomedical:** - INFOMEDX=IMEDX+ITHEX+IOTHX+IAGDEX+CNLMDX+CNLDEX - **Education and Counseling-lifestyle/psychosocial:** - INFOPSYX=ILSPSX+IAGSOX+CNLLSX+CNLSOX - **Facilitation and Patient Activation:** - PARTNERX=ASKOX+ASKPX+ASKRX+ASKUX+BCX+CHECX - **Rapport-building/positive:** - POSX=LAUGX+APPX+COMPX+AGREX - **Rapport-building/emotional:** - EMOX=EMPX+LEGITX+CONX+ROX+PARTX+SDISX - **Rapport-building/negative:** - NEGX=DISX+CRITX - **Rapport-building/social:** - CHITX=PERSX | - **Question-asking-biomedical:** - MEDQUEZ=QMEDZ+QTHEZ+QOTHZ+BIDZ+QAGDEZ - **Question-asking-lifestyle/psychosocial**: - PSYQUEZ=QLSPSZ+QAGSOZ - **Information giving-biomedical:** - INFOMEDZ=IMEDZ+ITHEZ+IOTHZ+IAGDEZ - **Information giving-lifestyle/psychosocial:** - INFOPSYZ=ILSPSZ+IAGSOZ - **Patient activation and engagement:** - PARTNERZ=ASKSZ+ASKRZ+ASKUZ+BCZ+CHECZ - **Rapport-building/positive:** - POSZ=LAUGZ+APPZ+COMPZ+AGREZ - Rapport-building/emotional: - EMOZ=EMPZ+LEGITZ+CONZ+ROZ - Rapport-building/negative: - NEGZ=DISZ+CRITZ - Rapport-building/social: - CHITZ=PERSZ |
| **Patient centeredness score** | **(PSYQUEX + INFOPSYX + EMOX + PSYQUEZ + PARTNERX + INFOPSYZ + EMOZ + MEDQUEZ )/ ( MEDQUEX + PROCX + INFOMEDZ + INFOMEDX )** |

**Fig D. Dendrogram and scree plots from hierarchical cluster analysis with practice as unit of analysis for WCC coding**

**
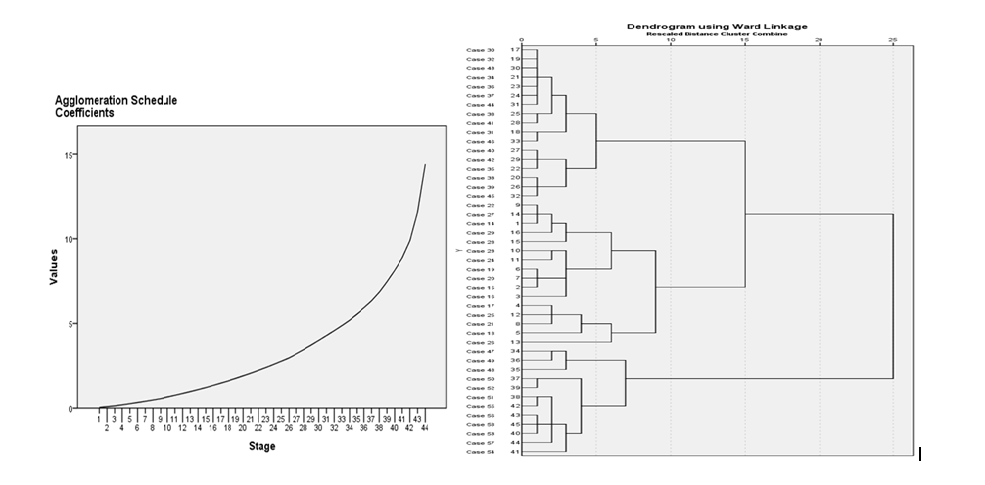
**

**Fig E. Relationship with patient centeredness scores with duration of consult and WCC coding**


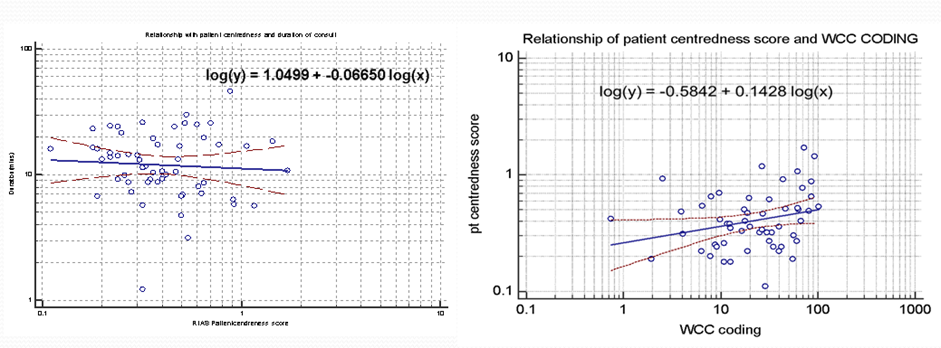


**Fig F. Histogram and normal P-P Plot of standardised residual dependent variable**

**
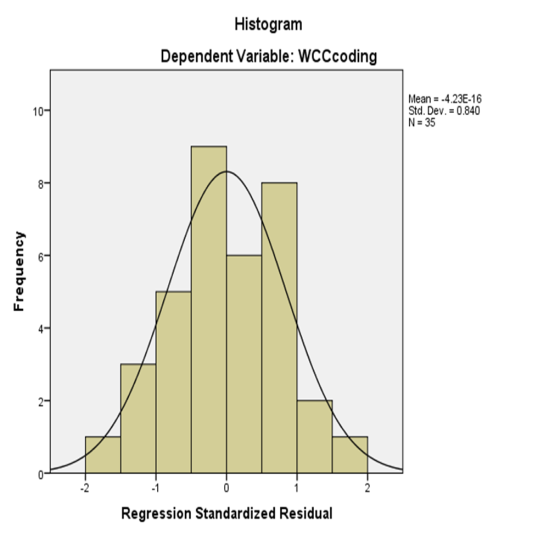

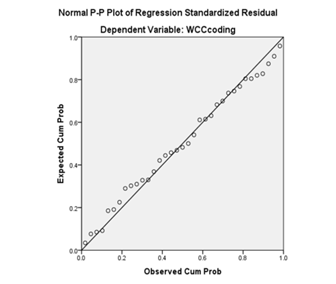
**
