# Supplementary material for: A case study of well child care visits at general practices in a region of disadvantage in Sydney
Source: PLoS One. 2018 Oct 11;13(10):e0205235. doi: 10.1371/journal.pone.0205235 (PMC6181326; doi:10.1371/journal.pone.0205235)
Supplement: S2 Appendix — (DOCX) [file pone.0205235.s002.docx]

**Fig A. Flow chart of data loss**

71 GP consults

with young children

21 consults

Audio poor quality for analysis

50 good quality records

Missing background demographic information

27 consults

Complete data for 44 consults

MVA VARIABLES=Previousvisits Agechildrenmonths Durationmins Purposeofvisit WCCcoding Firstborn

Matbirthcountry Mateducationstatus ContunityGPgroup

/MAXCAT=25

/CATEGORICAL=Firstborn Matbirthcountry Mateducationstatus ContunityGPgroup

/CROSSTAB PERCENT=5

/MPATTERN

/REGRESSION(TOLERANCE=0.001 FLIMIT=4.0 ADDTYPE=RESIDUAL).

**MVA (Missing Variable Analysis)**

| **Notes** | | |
| --- | --- | --- |
| Output Created | | 14-NOV-2016 22:19:15 |
| Comments | |  |
| Input | Data | C:\Users\gargpa\Desktop\Two more PhD papers\RIAS final coding and file\REGRESSION ANALYSIS FILE.sav |
|  | Active Dataset | DataSet2 |
|  | Filter | <none> |
|  | Weight | <none> |
|  | Split File | <none> |
|  | N of Rows in Working Data File | 71 |
| Syntax | | MVA VARIABLES=Previousvisits Agechildrenmonths Durationmins Purposeofvisit WCCcoding Firstborn  Matbirthcountry Mateducationstatus ContunityGPgroup  /MAXCAT=25  /CATEGORICAL=Firstborn Matbirthcountry Mateducationstatus ContunityGPgroup  /CROSSTAB PERCENT=5  /MPATTERN  /REGRESSION(TOLERANCE=0.001 FLIMIT=4.0 ADDTYPE=RESIDUAL). |
| Resources | Processor Time | 00:00:00.02 |
|  | Elapsed Time | 00:00:00.03 |

| **Univariate Statistics** | | | | | | | |
| --- | --- | --- | --- | --- | --- | --- | --- |
|  | N | Mean | Std. Deviation | Missing | | No. of Extremes^a^ | |
|  |  |  |  | Count | Percent | Low | High |
| Previousvisits | 60 | 8.50 | 6.922 | 11 | 15.5 | 0 | 2 |
| Agechildrenmonths | 68 | 22.47 | 18.238 | 3 | 4.2 | 0 | 1 |
| Durationmins | 66 | 13.98 | 6.923 | 5 | 7.0 | 0 | 1 |
| Purposeofvisit | 68 | 2.65 | .989 | 3 | 4.2 | 0 | 0 |
| WCCcoding | 66 | 31.9921 | 26.09870 | 5 | 7.0 | 0 | 0 |
| Firstborn | 59 |  |  | 12 | 16.9 |  |  |
| Matbirthcountry | 59 |  |  | 12 | 16.9 |  |  |
| Mateducationstatus | 59 |  |  | 12 | 16.9 |  |  |
| ContunityGPgroup | 57 |  |  | 14 | 19.7 |  |  |

| a. Number of cases outside the range (Q1 - 1.5*IQR, Q3 + 1.5*IQR). |
| --- |

| **Summary of Estimated Means** | | | | | |
| --- | --- | --- | --- | --- | --- |
|  | Previousvisits | Agechildrenmonths | Durationmins | Purposeofvisit | WCCcoding |
| All Values | 8.50 | 22.47 | 13.98 | 2.65 | 31.9921 |
| Regression | 8.60 | 23.18 | 14.09 | 2.65 | 32.5355 |

| **Summary of Estimated Standard Deviations** | | | | | |
| --- | --- | --- | --- | --- | --- |
|  | Previousvisits | Agechildrenmonths | Durationmins | Purposeofvisit | WCCcoding |
| All Values | 6.922 | 18.238 | 6.923 | .989 | 26.09870 |
| Regression | 6.814 | 19.302 | 6.836 | .984 | 25.95077 |

**Crosstabulations of Categorical Versus Indicator Variables**

| **Firstborn** | | | | | | |
| --- | --- | --- | --- | --- | --- | --- |
|  | | | Total | 1 | 2 | Missing |
|  |  |  |  |  |  | SysMis |
| Previousvisits | Present | Count | 60 | 18 | 40 | 2 |
|  |  | Percent | 84.5 | 100.0 | 97.6 | 16.7 |
|  | Missing | % SysMis | 15.5 | .0 | 2.4 | 83.3 |
| Durationmins | Present | Count | 66 | 17 | 39 | 10 |
|  |  | Percent | 93.0 | 94.4 | 95.1 | 83.3 |
|  | Missing | % SysMis | 7.0 | 5.6 | 4.9 | 16.7 |
| WCCcoding | Present | Count | 66 | 15 | 40 | 11 |
|  |  | Percent | 93.0 | 83.3 | 97.6 | 91.7 |
|  | Missing | % SysMis | 7.0 | 16.7 | 2.4 | 8.3 |
| Matbirthcountry | Present | Count | 59 | 17 | 39 | 3 |
|  |  | Percent | 83.1 | 94.4 | 95.1 | 25.0 |
|  | Missing | % SysMis | 16.9 | 5.6 | 4.9 | 75.0 |
| Mateducationstatus | Present | Count | 59 | 18 | 39 | 2 |
|  |  | Percent | 83.1 | 100.0 | 95.1 | 16.7 |
|  | Missing | % SysMis | 16.9 | .0 | 4.9 | 83.3 |
| ContunityGPgroup | Present | Count | 57 | 17 | 39 | 1 |
|  |  | Percent | 80.3 | 94.4 | 95.1 | 8.3 |
|  | Missing | % SysMis | 19.7 | 5.6 | 4.9 | 91.7 |

| Indicator variables with less than 5% missing are not displayed. | | | | | | |
| --- | --- | --- | --- | --- | --- | --- |
| **Matbirthcountry** | | | | | | |
|  | | | Total | 1 | 2 | Missing |
|  |  |  |  |  |  | SysMis |
| Previousvisits | Present | Count | 60 | 43 | 15 | 2 |
|  |  | Percent | 84.5 | 100.0 | 93.8 | 16.7 |
|  | Missing | % SysMis | 15.5 | .0 | 6.3 | 83.3 |
| Durationmins | Present | Count | 66 | 41 | 15 | 10 |
|  |  | Percent | 93.0 | 95.3 | 93.8 | 83.3 |
|  | Missing | % SysMis | 7.0 | 4.7 | 6.3 | 16.7 |
| WCCcoding | Present | Count | 66 | 40 | 15 | 11 |
|  |  | Percent | 93.0 | 93.0 | 93.8 | 91.7 |
|  | Missing | % SysMis | 7.0 | 7.0 | 6.3 | 8.3 |
| Firstborn | Present | Count | 59 | 42 | 14 | 3 |
|  |  | Percent | 83.1 | 97.7 | 87.5 | 25.0 |
|  | Missing | % SysMis | 16.9 | 2.3 | 12.5 | 75.0 |
| Mateducationstatus | Present | Count | 59 | 42 | 15 | 2 |
|  |  | Percent | 83.1 | 97.7 | 93.8 | 16.7 |
|  | Missing | % SysMis | 16.9 | 2.3 | 6.3 | 83.3 |
| ContunityGPgroup | Present | Count | 57 | 42 | 13 | 2 |
|  |  | Percent | 80.3 | 97.7 | 81.3 | 16.7 |
|  | Missing | % SysMis | 19.7 | 2.3 | 18.8 | 83.3 |

| Indicator variables with less than 5% missing are not displayed. |
| --- |

| **Mateducationstatus** | | | | | | |
| --- | --- | --- | --- | --- | --- | --- |
|  | | | Total | 1 | 2 | Missing |
|  |  |  |  |  |  | SysMis |
| Previousvisits | Present | Count | 60 | 19 | 40 | 1 |
|  |  | Percent | 84.5 | 100.0 | 100.0 | 8.3 |
|  | Missing | % SysMis | 15.5 | .0 | .0 | 91.7 |
| Durationmins | Present | Count | 66 | 17 | 39 | 10 |
|  |  | Percent | 93.0 | 89.5 | 97.5 | 83.3 |
|  | Missing | % SysMis | 7.0 | 10.5 | 2.5 | 16.7 |
| WCCcoding | Present | Count | 66 | 18 | 37 | 11 |
|  |  | Percent | 93.0 | 94.7 | 92.5 | 91.7 |
|  | Missing | % SysMis | 7.0 | 5.3 | 7.5 | 8.3 |
| Firstborn | Present | Count | 59 | 19 | 38 | 2 |
|  |  | Percent | 83.1 | 100.0 | 95.0 | 16.7 |
|  | Missing | % SysMis | 16.9 | .0 | 5.0 | 83.3 |
| Matbirthcountry | Present | Count | 59 | 19 | 38 | 2 |
|  |  | Percent | 83.1 | 100.0 | 95.0 | 16.7 |
|  | Missing | % SysMis | 16.9 | .0 | 5.0 | 83.3 |
| ContunityGPgroup | Present | Count | 57 | 19 | 37 | 1 |
|  |  | Percent | 80.3 | 100.0 | 92.5 | 8.3 |
|  | Missing | % SysMis | 19.7 | .0 | 7.5 | 91.7 |

| Indicator variables with less than 5% missing are not displayed. |
| --- |

| **ContunityGPgroup** | | | | | | |
| --- | --- | --- | --- | --- | --- | --- |
|  | | | Total | 1 | 2 | Missing |
|  |  |  |  |  |  | SysMis |
| Previousvisits | Present | Count | 60 | 47 | 10 | 3 |
|  |  | Percent | 84.5 | 100.0 | 100.0 | 21.4 |
|  | Missing | % SysMis | 15.5 | .0 | .0 | 78.6 |
| Durationmins | Present | Count | 66 | 44 | 10 | 12 |
|  |  | Percent | 93.0 | 93.6 | 100.0 | 85.7 |
|  | Missing | % SysMis | 7.0 | 6.4 | .0 | 14.3 |
| WCCcoding | Present | Count | 66 | 44 | 9 | 13 |
|  |  | Percent | 93.0 | 93.6 | 90.0 | 92.9 |
|  | Missing | % SysMis | 7.0 | 6.4 | 10.0 | 7.1 |
| Firstborn | Present | Count | 59 | 47 | 9 | 3 |
|  |  | Percent | 83.1 | 100.0 | 90.0 | 21.4 |
|  | Missing | % SysMis | 16.9 | .0 | 10.0 | 78.6 |
| Matbirthcountry | Present | Count | 59 | 46 | 9 | 4 |
|  |  | Percent | 83.1 | 97.9 | 90.0 | 28.6 |
|  | Missing | % SysMis | 16.9 | 2.1 | 10.0 | 71.4 |
| Mateducationstatus | Present | Count | 59 | 46 | 10 | 3 |
|  |  | Percent | 83.1 | 97.9 | 100.0 | 21.4 |
|  | Missing | % SysMis | 16.9 | 2.1 | .0 | 78.6 |

| Indicator variables with less than 5% missing are not displayed. | | | | | | | | | |  |  |
| --- | --- | --- | --- | --- | --- | --- | --- | --- | --- | --- | --- |
| **Missing Patterns (cases with missing values)** | | | | | | | | | | | |
| Case | # Missing | % Missing | Missing and Extreme Value Patterns^a^ | | | | | | | | |
|  |  |  | Agechildrenmonths | Purposeofvisit | Durationmins | WCCcoding | Previousvisits | Mateducationstatus | Matbirthcountry | | Firstborn |
| 9 | 1 | 11.1 |  |  |  |  |  |  |  | | S |
| 2 | 2 | 22.2 |  |  |  |  |  |  |  | | S |
| 23 | 1 | 11.1 |  |  |  |  |  |  |  | |  |
| 47 | 1 | 11.1 |  |  |  |  |  |  |  | |  |
| 20 | 1 | 11.1 | S |  |  |  |  |  |  | |  |
| 44 | 1 | 11.1 | S |  |  |  |  |  |  | |  |
| 30 | 1 | 11.1 |  |  |  | S |  |  |  | |  |
| 39 | 1 | 11.1 |  |  |  | S |  |  |  | |  |
| 45 | 1 | 11.1 |  |  |  | S |  |  |  | |  |
| 66 | 1 | 11.1 |  |  |  | S |  |  |  | |  |
| 33 | 1 | 11.1 |  |  | S |  |  |  |  | |  |
| 37 | 1 | 11.1 |  |  | S |  |  |  |  | |  |
| 21 | 2 | 22.2 |  | S | S |  |  |  |  | |  |
| 40 | 1 | 11.1 |  | S |  |  |  |  |  | |  |
| 48 | 1 | 11.1 |  |  |  |  |  | S |  | |  |
| 61 | 1 | 11.1 |  |  |  |  |  |  | S | |  |
| 65 | 1 | 11.1 |  |  |  |  |  |  | S | |  |
| 4 | 4 | 44.4 |  |  |  |  | S | S | S | |  |
| 14 | 5 | 55.6 |  |  |  |  | S | S | S | | S |
| 34 | 5 | 55.6 |  |  |  |  | S | S | S | | S |
| 53 | 5 | 55.6 |  |  |  |  | S | S | S | | S |
| 7 | 5 | 55.6 |  |  |  |  | S | S | S | | S |
| 63 | 5 | 55.6 |  |  |  |  | S | S | S | | S |
| 64 | 5 | 55.6 |  |  |  |  | S | S | S | | S |
| 18 | 5 | 55.6 |  |  |  |  | S | S | S | | S |
| 15 | 4 | 44.4 |  |  |  |  | S | S |  | | S |
| 22 | 7 | 77.8 |  |  | S | S | S | S | S | | S |
| 17 | 8 | 88.9 | S | S | S |  | S | S | S | | S |

| **Missing Patterns (cases with missing values)** | |
| --- | --- |
| Case | Missing and Extreme Value Patterns |
|  | ContunityGPgroup |
| 9 |  |
| 2 | S |
| 23 | S |
| 47 | S |
| 20 |  |
| 44 |  |
| 30 |  |
| 39 |  |
| 45 |  |
| 66 |  |
| 33 |  |
| 37 |  |
| 21 |  |
| 40 |  |
| 48 |  |
| 61 |  |
| 65 |  |
| 4 | S |
| 14 | S |
| 34 | S |
| 53 | S |
| 7 | S |
| 63 | S |
| 64 | S |
| 18 | S |
| 15 | S |
| 22 | S |
| 17 | S |

| - indicates an extreme low value, while + indicates an extreme high value. The range used is (Q1 - 1.5*IQR, Q3 + 1.5*IQR). |
| --- |
| a. Cases and variables are sorted on missing patterns. |

**Regression Estimated Statistics**

| **Regression Means^a^** | | | | |
| --- | --- | --- | --- | --- |
| Previousvisits | Agechildrenmonths | Durationmins | Purposeofvisit | WCCcoding |
| 8.60 | 23.18 | 14.09 | 2.65 | 32.5355 |

| a. Residual of a randomly chosen case is added to each estimate. |
| --- |

| **Regression Covariances^a^** | | | | | |
| --- | --- | --- | --- | --- | --- |
|  | Previousvisits | Agechildrenmonths | Durationmins | Purposeofvisit | WCCcoding |
| Previousvisits | 46.426 |  |  |  |  |
| Agechildrenmonths | 51.015 | 372.580 |  |  |  |
| Durationmins | -5.865 | -21.438 | 46.735 |  |  |
| Purposeofvisit | 1.763 | 7.573 | -2.376 | .967 |  |
| WCCcoding | -48.462 | -189.375 | 69.470 | -12.794 | 673.44223 |

| a. Residual of a randomly chosen case is added to each estimate. |
| --- |

| **Regression Correlations^a^** | | | | | |
| --- | --- | --- | --- | --- | --- |
|  | Previousvisits | Agechildrenmonths | Durationmins | Purposeofvisit | WCCcoding |
| Previousvisits | 1 |  |  |  |  |
| Agechildrenmonths | .388 | 1 |  |  |  |
| Durationmins | -.126 | -.162 | 1 |  |  |
| Purposeofvisit | .263 | .399 | -.353 | 1 |  |
| WCCcoding | -.274 | -.378 | .392 | -.501 | 1 |

| a. Residual of a randomly chosen case is added to each estimate. |
| --- |
