# Supplementary material for: A case study of well child care visits at general practices in a region of disadvantage in Sydney
Source: PLoS One. 2018 Oct 11;13(10):e0205235. doi: 10.1371/journal.pone.0205235 (PMC6181326; doi:10.1371/journal.pone.0205235)
Supplement: S3 Appendix — Fig A Flow chart of Data loss Missing Variable Analysis. (DOCX) [file pone.0205235.s003.docx]

**ROTERS INTERACTION ANALYSIS SYSTEM, CODER RELIABILITY (n=6)**

Correlations: Doctor talk to parent/pt

***Average correlation on categories with a mean >1.0 = .885***

***Average correlation on categories with a mean >2.0 = .886***

persd .250**

laugd .765*

appd 1.000*

compd 1.000** .

disd 1.000**

critd .

empd 1.000**

legitd .

cond .919

rod .904

partd .

sdisd .

imedd .968

ithed .809*

ilspsd 1.000**

iagded .878**

iagsod .

iothd .

agred .873

bcd .976

checd .860

trand .816

orid .975

cmedd .922

cthed 1.000

clspsd 1.000**

cagded .971**

cagsod .632**

cothd .

omedd .774

otherd .999**

olspsd 1.000**

oagded .197**

oagsod 1.000**

oothd .

askod .894**

askpd .

askrd .

askud .869

bidd .

cnlmdd .663

cnllsd 1.000**

cnlded .948*

cnlsod .

# Correlations: Parent/pt to doctor

***Average correlation on categories with a mean >1.0 = .871***

***Average correlation on categories with a mean > 2.0 = .932***

persp .250**

laugp .828*

appp .927*

comp .

disp .

critp .

empp .

legitp .

conp .938

rop .790*

imedp .954

ithep .999

ilspsp .889*

iagdep .986

iagsop .632**

iothp .

agrep .963

checp .750

tranp .557*

orip .

qmedp 1.000**

qthep .333**

qlspsp .

qagdep .

qagsop .

qothp .

asksp 1.000**

askrp .

askup .

bidp 1.000**

* = mean 1.0-2.0

** =mean <1.0

RELIABILITY/AFFECT RATINGS (n=6)

Coder agreements/

possible agreements % agreement

ANGD 6/6 100.0

ANXD 6/6 100.0

DOMD 6/6 100.0

INTD 6/6 100.0

WARMD 6/6 100.0

ENGAGD 6/6 100.0

SYMD 6/6 100.0

HURD 6/6 100.0

RESPTD 6/6 100.0

INTERD 6/6 100.0

ANG2 6/6 100.0

ANX2 6/6 100.0

DOM2 6/6 100.0

INT2 6/6 100.0

WARM2 6/6 100.0

ENGAG2 6/6 100.0

SYM2 6/6 100.0

RESPT2 6/6 100.0

ANGP 6/6 100.0

ANXP 6/6 100.0

DEPRP 6/6 100.0

DISTP 6/6 100.0

DOMP 6/6 100.0

INTP 6/6 100.0

WARMP 6/6 100.0

ENGAGP 6/6 100.0

SYMP 6/6 100.0

RESPTP 6/6 100.0

INTERP 6/6 100.0

ANGC 6/6 100.0

ANXC 6/6 100.0

DOMC 6/6 100.0

INTC 6/6 100.0

WARMC 6/6 100.0

ENGAGC 6/6 100.0

SYMC 6/6 100.0

RESPTC 6/6 100.0
